# TCF3 as a multidimensional biomarker: oncogenicity, genomic alterations, and immune landscape in pan-cancer analysis: TCF3 as a pan-cancer multidimensional biomarker

**DOI:** 10.3724/abbs.2024126

**Published:** 2024-08-27

**Authors:** Huiling Nie, Yang Yu, Siqi Zhou, Yue Xu, Xi Chen, Xun Qin, Zhangyu Liu, Jiayu Huang, Hailiang Zhang, Jin Yao, Qin Jiang, Bingbing Wei, Xiaojian Qin

**Affiliations:** 1 The Affiliated Eye Hospital and the Fourth School of Clinical Medicine Nanjing Medical University Nanjing 210004 China; 2 Department of Urology Fudan University Shanghai Cancer Center; Department of Oncology Shanghai Medical College Fudan University Shanghai 200032 China; 3 Department of Ophthalmology the Fourth Affiliated Hospital of Soochow University Suzhou 215002 China; 4 Department of Urology the Affiliated Wuxi People’s Hospital of Nanjing Medical University Wuxi 214023 China

**Keywords:** TCF/LEF family, TCF3, pan-cancer analysis, prognosis, tumor microenvironment (TME), epithelial-mesenchymal transition (EMT)

## Abstract

Transcription factor 3 (TCF3), a pivotal member of the TCF/LEF family, plays a critical role in tumorigenesis. Nonetheless, its impact on the tumor microenvironment (TME) and cancer phenotypes remains elusive. We perform an exhaustive analysis of TCF3 expression, DNA variation profiles, prognostic implications, and associations with the TME and immunological aspects. This study is based on a large-scale pan-cancer cohort, encompassing over 17,000 cancer patients from multiple independent datasets, validated by *in vitro* assays. Our results show that TCF3/4/7 exhibits differential expression patterns between normal and tumor tissues across pan-cancer analyses. Mutational analysis of TCF3 across diverse cancer types reveals the highest alteration rates in biliary tract cancer. Additionally, mutations and single nucleotide variants in TCF3/4/7 are found to exert varied effects on patient prognosis. Importantly, TCF3 emerges as a robust predictor of survival across all cancer cohorts and among patients receiving immune checkpoint inhibitors. Elevated TCF3 expression is correlated with more aggressive cancer subtypes, as validated by immunohistochemistry and diverse cohort data. Furthermore, TCF3 expression is positively correlated with intratumoral heterogeneity and angiogenesis. *In vitro* investigations demonstrate that TCF3 is involved in epithelial-mesenchymal transition, migration, invasion, and angiogenesis. These effects are likely mediated through the interaction of TCF3 with the NF-κB/MMP2 pathway, which is modulated by IL-17A in human uveal melanoma MUM2B cells. This study elucidates, for the first time, the significant associations of TCF3 with DNA variation profiles, prognostic outcomes, and the TME in multiple cancer contexts. TCF3 holds promise as a molecular marker for diagnosis and as a potential target for novel therapeutic strategies, particularly in uveal melanoma.

## Introduction

The mammalian TCF/LEF family consists of four nuclear factors, namely, TCF7, LEF1, TCF7L1, and TCF7L2 (also referred to as TCF1, LEF1, TCF3, and TCF4, respectively) [1]. These proteins share common structural features and are typically co-expressed in an overlapping manner. High mobility group (HMG) box transcription factor 3 (TCF3) is a 63 kDa member of the TCF/LEF family. Human TCF3 has a full length of 588 amino acids, including an HMG box DNA binding domain (aa 346‒414). There is 95% amino acid sequence homology between human TCF3 and mouse TCF3 [ 1, 2]. The TCF/LEF family of proteins, with TCF-3 as a notable member, has garnered substantial attention in the field of cancer research due to its pivotal role in tumorigenesis. From an academic standpoint, progress in understanding TCF3 and its effects on tumor biology has revealed its potential clinical value and implications for therapeutic interventions [3].

In adult skin, TCF3 is naturally expressed in the bulge region of hair follicles and is considered a niche for multipotent stem cells (SCs) [4]. TCF3 is reportedly present in embryonic skin progenitor cells in mice, and its reactivation is associated with increased expressions of differentiation-inhibiting genes in epidermal cells, suggesting that TCF3 may function to maintain skin multipotent stem cells in an undifferentiated state [5]. Pereira *et al*. [4] reported that TCF3 impairs the self-renewal of embryonic stem cells by suppressing the transcription of the *Nanog* gene. Functionally, TCF proteins (TCF1, TCF3, TCF4, and LEF1 in mammals) act as DNA-binding transcriptional regulatory factors in the canonical Wnt signaling pathway. TCF3 interacts with DNA through a highly conserved HMG domain and an N-terminal β-catenin-binding domain. It functions as a repressor in the absence of β-catenin and as an activator in the presence of β-catenin [6]. Thus, it is important to investigate the mechanisms underlying drug resistance and discover new therapeutic targets for the TCF family.

It was estimated that approximately 4,824,700 new cancer cases and 2,574,200 new cancer deaths occurred in China in 2022 [7]. The tumor microenvironment (TME) refers to the local environment in which tumor initiation and progression occur. It primarily encompasses tumor cells, immune cells, stromal cells, and their secreted cytokines, as well as the extracellular matrix formed by their interactions [8]. The phenomenon of immune infiltration encompasses the presence of all immune cells within the TME, including but not limited to T cells, macrophages, natural killer (NK) cells, myeloid-derived suppressor cells (MDSCs), mast cells, neutrophils, B cells, dendritic cells (DCs), and tertiary lymphoid structures (TLSs) [ 9‒ 11]. Notably, certain cell types within the TME may exhibit both anti-tumor and pro-tumor effects [12]. For instance, macrophages can be simplified into two categories: M1 macrophages, which are associated with acute inflammation and anti-tumor activity, and M2 macrophages, which are recruited for pro-carcinogenic chronic inflammation [13]. This dual role of specific cell types within the TME highlights the complexity of the interactions that influence tumor behavior and the immune response. Therefore, achieving effective anti-cancer therapies requires overcoming the “immune-cold” phenotype of the TME [14].

Notably, TME plays a decisive role in the heterogeneity of tumors, as tumor cells and their surrounding microenvironment are interdependent and mutually influential. Tumor cells release extracellular signals that impact the TME, inducing immune tolerance [15]. Simultaneously, immune cells infiltrating the TME can eliminate tumor cells, preventing their proliferation and metastasis. The TME encompasses two distinct immune suppression mechanisms: intrinsic immune suppression, possibly induced by genetic alterations within the tumor, involving the activation of various oncogenic pathways leading to the development of cold tumors, and local adaptive immune suppression, resulting in a highly T-cell-infiltrated hot tumor environment, providing a favorable setting for effective immune checkpoint inhibitor (ICI)-based monotherapy or combination therapy [ 16, 17]. Strategies must be developed to enhance the efficacy of ICIs by modulating the TME and shifting the tumor phenotype from cold to hot.

The characteristics of a cold or hot tumor TME include the presence of sufficient immune cells, immune factors, or other immunomodulatory elements. In the immune microenvironment of cold tumors, activation of immune checkpoint (ICP) inhibitory pathways such as PD-1/PD-L1 and CTLA-4 leads to fewer immune cells, such as T cells and NK cells, and an abundance of immune inhibitory factors, such as interleukin-10 (IL-10), IL-17, and transforming growth factor-beta (TGF-β), thereby suppressing the proliferation and activation of immune cells [ 18, 19]. Additionally, surface molecules on tumor cells, such as PD-L1, inhibit the activation of immune cells. In contrast, the immune microenvironment of hot tumors is enhanced through the activation of immune stimulatory pathways such as CD28/B7 and CD40/CD40L, resulting in a higher number of immune cells, including T cells, NK cells, and DCs [20]. Fewer immune inhibitory factors are present, leading to a robust immune response. Immune activation factors released by tumor cells stimulate the proliferation and activation of immune cells, while surface immune checkpoint molecules such as PD-L1 on tumor cells are targeted and eliminated by immune cells [21].

In this study, we focused on elucidating the potential impact of the TCF family on clinical malignant features and the TME in various cancers. Our hypothesis posited that, in practical clinical settings, TCFs could serve as valuable tools for a comprehensive assessment of prognostic patterns and the specific infiltration of immune cells within the TME among patients. This approach enables the identification of distinct immunophenotypes, facilitating the development of effective clinical treatment strategies for different cancer types.

## Materials and Methods

### Cancer sample collection and study cohorts

The RNA-seq, copy number variation, mutation, clinical, and survival data of 10,228 pan-cancer patients were obtained from The Cancer Genome Atlas (TCGA, https://portal.gdc.cancer.gov) database [22]. Moreover, the normal tissue sample data were obtained from the GTEx V8 version ( https://gtexportal.org/home/datasets) and complete with detailed clinical and pathological annotations of the participants, as provided in the official GTEx annotation. Additionally, more than 10,000 cancer samples featuring DNA variation profiles from 378 separate cohorts were selected via the cBioPortal for Cancer Genomics ( http://www.cbioportal.org/) for the purpose of examining the influence of genomic alterations in TCF4, TCF3, and TCF7 in cancer research. The Gene Expression Omnibus (GEO) database contains RNA sequencing data along with subsequent survival information for 875 patients with gastric cancer and 1925 participants with breast cancer. A total of 933 individuals receiving ICIs were also enrolled in this study, including 73 patients with bladder cancer, 348 subjects with urothelial cancer, 28 patients with glioblastoma, and 397 participants with breast cancer, all of which were included in the analysis. Furthermore, 70 KIRC (kidney renal clear cell carcinoma) samples from a real-world cohort at Fudan University Shanghai Cancer Center (FUSCC, Shanghai) were used for further experimental validation. The RNA expression levels were expressed in various formats, such as Fragments Per Kilobase of Transcript Per Million mapped reads (FPKM), Transcripts Per Million (TPM), Reads Per Kilobase of transcript per Million mapped reads (RPKM), and RNA-Seq by Expectation Maximization (RSEM), and the formats used for each result were consistent. Gene expression data for Uveal Melanoma (UVM) were extracted from the TCGA database by aligning the raw RNA-seq reads to the human reference genome. Quantification of transcript abundances was then performed using distinct normalization methods, resulting in datasets presented in FPKM, TPM, RPKM, and RSEM formats, each providing unique insights into gene expression levels and variations in 79 UVM samples from the TCGA database. All of the study designs and test procedures were performed in accordance with the Helsinki Declaration II. The ethics approval and participation consent of this study were approved and agreed upon by the Ethics Committee of Fudan University Shanghai Cancer Center (FUSCC, Shanghai, China).

### Differential expression and survival analysis

To illustrate the statistical relevance of the variations in TCF4, TCF3, and TCF7 expression between tumor and normal tissues, an unpaired Student’s *t* test was utilized to assess the disparities across different cancer types. The Kaplan-Meier method, along with 95% confidence intervals (95% CIs) and log-rank tests, was used to determine the impact of the expression levels of TCF4, TCF3, and TCF7 on progression-free survival (PFS), disease-specific survival (DSS), and overall survival (OS). Survival outcomes were analyzed for distinct expression cohorts and further stratified into subgroups based on tumor microenvironment infiltration characteristics using the Kaplan-Meier Plotter tool ( https://kmplot.com/analysis/). To pinpoint the most informative predictors for constructing a prognostic nomogram, both univariate and multivariate Cox regression analyses were conducted. The forest plot was used to show the *P* value, hazard ratio (HR), and 95% confidence interval (CI) of each variable through the “forestplot” R package [23].

### Abundance and frequency of mutations

To explore the role of TCF4, TCF3, and TCF7 across cancers, we scrutinized their mutation abundance and frequency across cancers via the cBioportal for Cancer Genomics ( http://www.cbioportal.org/) [24]. Cancer samples with available DNA information, including information on mutations, copy number variations (CNVs), and somatic number variations (SNVs), and data from several independently curated nonredundant cohorts were collected. Postcuration, the mutation frequency of key genes related to the variable expression of TCF4, TCF3, and TCF7 was examined. Genes significantly upregulated in samples with TCF4, TCF3, and TCF7 alterations compared to those without alterations were identified and verified using the Limma R package [25].

### Relationship between TCF3 expression and immunity

We analyzed the co-expression of TCF3 and immune-related genes, specifically genes encoding major histocompatibility complex (MHC) and immune activation, immunosuppressive, chemokine, and chemokine receptor proteins. Additionally, the abundances of 22 immune cell subtypes in cancer samples were assessed with the CIBERSORT algorithm in R software [26]. In addition, the Tumor Immune Estimation Resource 2.0 (TIMER 2.0; http://timer.cistrome.org/) was used to assess the relationship between the abundance of tumor-infiltrating immune cells and TCF3 expression through Pearson’s test [27]. The correlations between TCF3 and each immune cell infiltration level were assessed based on the R software packages “ggplot2” and “ggpubr”. Next, we evaluated the immune score, stromal score, ESTIMATE score, and tumor purity of each ccRCC sample with the ESTIMATE algorithm [28].

### Immunohistochemistry staining

Immunohistochemical analysis (IHC) was performed using an anti-TCF3 antibody (PA5-20900; Thermo Fisher Scientific, Waltham, USA) at a concentration of 5 μg/mL. Tissue samples were initially fixed in 10% neutral buffered formalin for 24 h at room temperature, followed by dehydration through a graded ethanol series (70%, 95%, and 100%), clearing with xylene, and embedding in paraffin. Sections of 4 μm thickness were cut using a microtome and mounted on microscope slides, then dried overnight at 37°C.

For staining, the paraffin-embedded sections were deparaffinized in xylene and rehydrated through decreasing concentrations of ethanol (100%, 95%, and 70%) and rinsed in phosphate-buffered saline (PBS). Antigen retrieval was performed by heating the sections in citrate buffer (pH 6.0) at 95°C for 20 min. After cooling to room temperature, the sections were incubated with 3% hydrogen peroxide in methanol for 10 min to quench endogenous peroxidase activity. Sections were then blocked with normal goat serum for 30 min to prevent nonspecific binding. The primary antibody, diluted to 5 μg/mL, was applied and incubated at 4°C overnight in a humidity chamber. After wash with PBS, the sections were incubated with a biotinylated secondary antibody (from the ABC kit, Beyotime Biotechnology, Shanghai, China) for 30 min, followed by incubation with the avidin-biotin complex solution for another 30 min.

The signal was developed using the 3,3′-diaminobenzidine (DAB) substrate kit (Beyotime Biotechnology) until the desired intensity was reached (typically 5‒10 min), and the sections were counterstained with hematoxylin for 1‒2 min. Finally, the slides were dehydrated through ascending alcohol concentrations, cleared in xylene, and mounted with coverslips. The degree of IHC staining was assessed based on the integration of staining intensity and density by two experienced and independent pathologists. The IHC score was evaluated on a scale from 0 to 12, where a score of 0 to 3 was defined as negative staining, and a score of 4 to 12 was defined as positive staining for each tissue sample.

### Association of TCF3 with tumor heterogeneity and functional enrichment analysis

The tumor heterogeneity score relied mainly on the MATH algorithm [29]. Subsequently, these heterogeneity scores were integrated with gene expression data from the samples to establish the Pearson correlation between them. The correlation between TCF3 and 14 cancer functional states was analyzed using single-cell sequence data from the “correlation plot” module of the CancerSEA website ( biocc. hrbmu.edu.cn/CancerSEA/home.jsp).

### Cell culture

The uveal melanoma cell line MUM2B was purchased from the American Type Culture Collection (ATCC, Manassas, USA). The cells were cultured in RPMI-1640 (Gibco, Carlsbad, USA) media supplemented with 10% fetal bovine serum (FBS; HyClone, Shanghai, China), 100 U/mL penicillin (Beyotime, Shanghai, China), and 100 μg/mL streptomycin (Gibco). All cells were maintained in a humidified atmosphere incubator with 5% CO _2_ at 37°C (Thermo Fisher Scientific).

### Cell transfection

MUM2B cells were transfected with double-stranded small interfering RNA (siRNA) in a 6-well plate using Lipofectamine 2000 reagent (RiboBio, Guangzhou, China) following the manufacturer’s protocol. siRNAs against TCF3 (si-TCF3) and the control were obtained from Sangon Biotech (Shanghai, China). The transfection dose used for each well was 10 μL of TCF3-siRNA1 (5′-AAGCAACAAAACATACACT-3′), siRNA-2 (5′-AGGAGAAGGAGGACGAGGAG-3′) or negative control RNAi (NC-siRNA, 5′- TTCTCCGAACGTGTCACGT-3′) mixed with RPMI-1640 media. The MUM2B cells were harvested after at least 24 h of transfection and incubated for subsequent experimental analysis.

### Western blot analysis

The cells were collected by scraping them into SDS sample buffer supplemented with a mixture of protease inhibitors and PhosSTOP phosphatase inhibitor (Roche, Basel, Switzerland). Western blot analysis was carried out according to the standard procedure [13]. The PVDF membranes (Millipore, Billerica, USA) were blocked using milk and then incubated with the following primary antibodies: anti-TCF3 (1:1000, PA5-20900; Thermo Fisher Scientific), anti-E-cadherin (1:1000, No. 3195; CST, Beverly, USA), anti-N-cadherin (1:1000, No. 13116; CST), anti-vimentin (1:1000, No. 5741; CST), anti-Snail (1:1000, No. 3879; CST), anti-TGF-β (1:1000, No. 3711; CST), anti-IL-17 (1:1000, No. 13838; CST), anti-MMP-2 (1:1000, No. 40994; CST), anti-MMP-9 (1:1000, No. 3852; CST), anti-P50 (1:1000, No. 3035; CST), anti-P65 (1:1000, No. 4764; CST), anti-VEGFA (1:1000, No. 50661; CST), and anti-GAPDH primary antibody (1:1000, No. 5174; CST). A HRP-conjugated goat anti-rabbit IgG (1:3000, ab205718; Abcam, Cambridge, UK) was used as the secondary antibody. Finally, the bands were visualized using ECL-plus™ western blotting chemiluminescence detection kits (BD Biosciences, Franklin Lakes, USA). GAPDH was used as an internal standard.

### Cell proliferation assay

Using an EdU assay kit (Ribobio), cell proliferation assay was conducted using an EdU assay kit (Ribobio) according to the manufacturer’s guidelines. The procedure was as follows: initially, 2×10 ^4^ cells were plated per well on coverslips in 24-well plates and left to settle overnight. After a 48-h incubation with MDL-800 [at 0 μM (DMSO vehicle only), 10 μM, or 25 μM], the cells were exposed to EdU-containing medium (50 μM final concentration) and incubated for an additional 2 h at 37°C. The cells were then fixed using 4% formaldehyde for 30 min, followed by permeabilization with 0.5% Triton X-100 for 10 min. An Apollo reaction cocktail was applied for 30 min at ambient temperature. To stain the DNA, Hoechst was used for 30 min, and the images were captured with a fluorescence microscope (ECLIPSE Ti; Nikon, Tokyo, Japan), with five random fields per well at 20× magnification. Image analysis was carried out using ImageJ software (National Institutes of Health, Bethesda, USA). The EdU incorporation rate was quantified as the percentage of EdU-positive cells relative to the total cell count in each visual field.

### Cell migration and invasion assay

In the cell invasion assay, 200 μL of serum-free medium containing 2×10 ^4^ transfected MUM2B cells was seeded into each Matrigel-coated upper chamber of a Transwell insert (Corning, New York, USA), while each lower chamber was filled with 800 μL of medium. After incubation for 12 h at 37°C, non-invasion cells in the upper chamber were removed using cotton swabs. Subsequently, the cells that invaded through the Matrigel-coated filters were fixed with 4% paraformaldehyde for 15 min and stained with 0.1% crystal violet. The invaded cells on the underside of the filters were quantified using a microscope (Nikon) across five random fields, and the entire procedure was replicated three times. The migration assay was the same as the invasion assay except that the upper chambers lacked the Matrigel coating (BD Biosciences).

### Matrigel tube formation

The tube formation assay was performed following the protocol described previously [30]. After 24 h of reoxygenation, the groups were starved overnight using cell culture medium containing 0.2% serum in preparation for the tube formation assay. The 24-well plates were coated with 250 μL of Matrigel (10 mg/mL; #354230; Corning) and incubated at 37°C for 30 min. A total of 100,000 MUM2B cells were resuspended in 300 μL of growth medium and added to each well, along with the same volume of vehicle PBS. After incubation in a CO _2_ incubator at 37 °C for 6 h, images were captured using an inverted microscope (Eclipse Ts2R; Nikon). The tube branch length was quantified using ImageJ software with the “Angiogenesis Analyser” plugin.

### Statistical analysis

All the statistical analyses were conducted utilizing SPSS version 23.0 (SPSS Software, Chicago, USA) [31], GraphPad Prism 8.0 (GraphPad Software, La Jolla, USA), R software (version 3.4.3), and online webtools [32]. Student’s *t* test was used for comparisons between two groups and one-way ANOVA was used for comparisons among three or more groups. All tests of hypotheses were performed using a two-sided approach, with a *P* value threshold of less than 0.05 denoting statistical significance.

## Results

### Differential expression of the TCF/LEF family in pan-cancer

**Figure 1:**
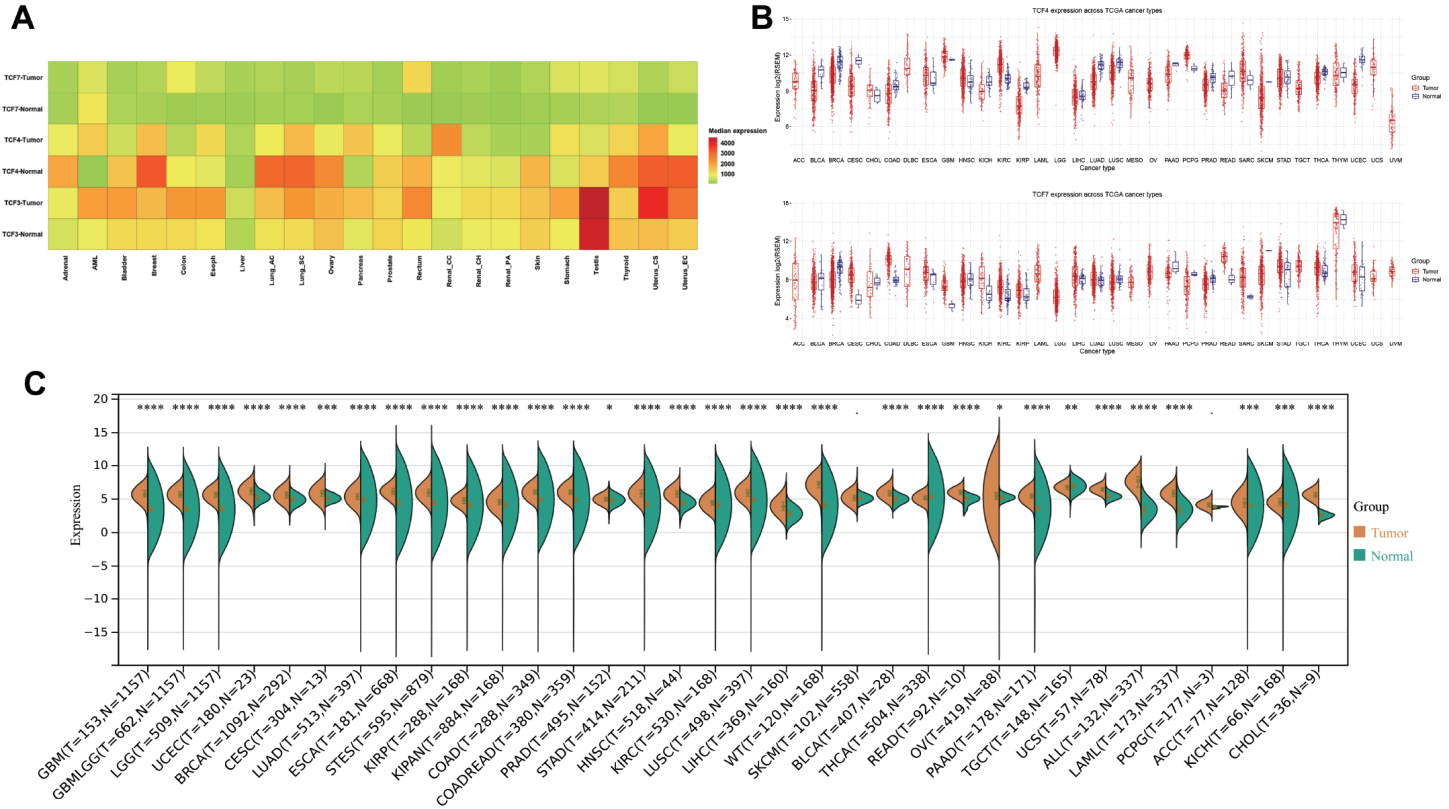
Figure 1 Differential expression of the TCF/LEF family members across cancers (A) The median levels of TCF7, TCF4, and TCF3, according to the FPKM and TPM expression values, in matched normal and tumor tissue samples from pancancer patients. (B) TCF4 and TCF7 expressions in normal and tumor tissues across TCGA cancer types, using the RSEM expression value. (C) TCF3 expression in normal and tumor tissues across TCGA cancer types. *P < 0.05, **P < 0.01, ***P < 0.001, ****P < 0.0001.

To investigate the transcription pattern of the TCF/LEF family, we first compared the median levels of TCF7, TCF4, and TCF3 in normal and tumor tissues from pan-cancer patients in the TCGA cohort using FPKM and TPM expression values ( Figure 1A). TCF7 was highly expressed in rectal cancer, while its expression levels did not differ significantly among the other tissues. The expression level of TCF4 decreased in most tumors except acute myeloid leukemia (AML), esophageal cancer, pancreatic cancer, renal cell carcinoma, and stomach cancer. TCF3 showed an overall upwards trend in tumor tissues, with the most significant increase observed in testis cancer, uterine carcinosarcoma, and uterine endometrial carcinoma. Next, we examined TCF4 and TCF7 expressions in normal and tumor tissues across TCGA cancer types, respectively, using the RSEM expression value ( Figure 1B). Similarly, the expression of TCF3 was also explored ( Figure 1C). We found that TCF3 was expressed at significantly higher levels in most cancers, especially in glioblastoma multiforme (GBM), breast invasive carcinoma (BRCA), colon adenocarcinoma (COAD), pancreatic adenocarcinoma (PAAD), acute lymphoblastic leukemia (ALL), acute myeloid leukemia (LAML), and cholangiocarcinoma (CHOL). Studies have reported the role of TCF3 in some of the above-mentioned tumors. Elevated TCF3 expression in human gliomas or poorly differentiated breast cancers may drive tumor progression via activation of the Akt/Erk signaling pathways, initiating tumor proliferation [ 33, 34]. Moreover, Taniue *et al*. [35] reported that the ASBEL-TCF3 complex is essential for the tumorigenic potential of colorectal cancer cells and plays a crucial role in tumorigenesis mediated by the Wnt/β-catenin pathway. Overall, the expressions of TCF7, TCF4, and TCF3, which are members of the TCF/LEF family of proteins, are significantly different between normal and tumor samples, indicating the potential involvement of TCF/LEF in the occurrence and progression of cancers.

### DNA variation landscape of TCF4, TCF3 and TCF7 in pan-cancer

**Figure 2:**
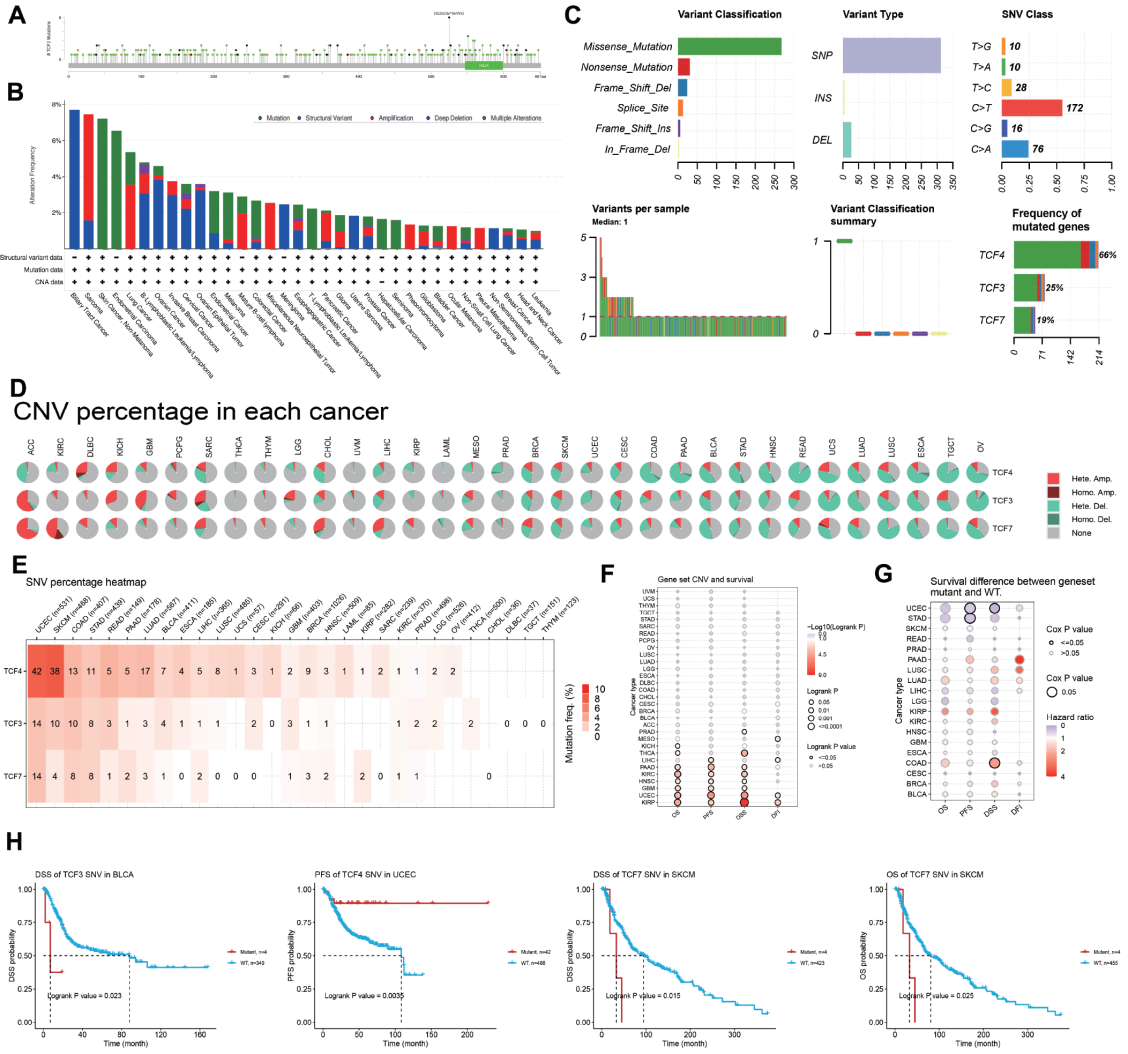
Figure 2 Mutation landscape of TCF4, TCF3, and TCF7 across cancers (A) Analysis of somatic mutations in TCF3 and its main mutation site. (B) Analysis of the alteration frequency of TCF3 across cancers. (C) The variant classification, variant type, and SNV category of TCF4, TCF3, and TCF7. (D) Association among heterozygosity and homozygous amplification and deletion of TCF4, TCF3, and TCF7. (E) The SNV frequencies of TCF4, TCF3, and TCF7. UCEC (42% for TCF4, 14% for TCF3 and TCF7), SKCM (38% for TCF4, 10% for TCF3, and 4% for TCF7), and COAD (13% for TCF4, 10% for TCF3, and 8% for TCF7). (F) The correlation between gene set CNVs and the prognosis of pancancer patients. (G) The correlation between gene set mutations and the prognosis of pancancer patients. (H) The prognostic value of the TCF3 SNV for DSS in BLCA patients (P = 0.023), the prognostic value of the TCF4 SNV for PFS in UCEC patients (P = 0.0035), the prognostic value of the TCF7 SNV for DSS in SKCM patients (P = 0.015), and the prognostic value of the TCF7 SNV for OS in SKCM patients (P = 0.025).

Subsequently, we performed a pan-cancer analysis of TCF3 mutations in the TCGA database. We discovered that the main mutation site of TCF3 ^MUT^ is in R525Gfs*18/W/G, mainly in the HLH domain protein ( Figure 2A). Then, we explored the mutation, structural variant, amplification, deep deletion, and multiple alteration frequencies of TCF3 ( Figure 2B). The results showed that TCF3 had the highest alteration frequency in biliary tract cancer, and deep deletions accounted for almost the entire proportion of alterations. The remaining patients had sarcoma non-melanoma skin cancer, and their mutation types were diverse. Overall, deep deletion and mutation of TCF3 were dominant in various types of tumors. Figure 2C summarized the variant classification, variant type, and SNV category of TCF4, TCF3, and TCF7. Next, we explored the CNV characteristics of TCF4, TCF3, and TCF7 ( Figure 2D). Heterozygous and homozygous amplification and deletion of TCF4, TCF3, and TCF7 were detected in pan-cancer patients. Then, we investigated the single nucleotide variant (SNV) frequency of TCF4, TCF3, and TCF7 in each tumor based on TCGA data ( Figure 2E). The results showed that the SNV frequencies of TCF4, TCF3, and TCF7 were highest in UCEC (42% for TCF4, 14% for TCF3 and TCF7), followed by skin cutaneous melanoma (SKCM) (38% for TCF4, 10% for TCF3, and 4% for TCF7) and colon adenocarcinoma (COAD) (13% for TCF4, 10% for TCF3, and 8% for TCF7). Furthermore, we analyzed the effects of CNVs in TCF3 on the prognosis of cancer patients ( Figure 2F). Gene set CNVs mostly correlated with the survival outcomes of pancreatic adenocarcinoma (PAAD), kidney renal clear cell carcinoma (KIRC), head and neck squamous cell carcinoma (HNSC), glioblastoma multiforme (GBM), uterine corpus endometrial carcinoma (UCEC), and kidney renal papillary cell carcinoma (KIRP) patients. Similarly, the effects of gene set mutations were also evaluated ( Figure 2G). The gene set mutant showed a high hazard ratio for DSS in patients with COAD, while it could be a protective factor against PFS in patients with UCEC and stomach adenocarcinoma (STAD). Finally, to investigate the impact of SNV profiles of different molecules on the survival of specific cancer types, we created relevant survival curve graphs ( Figure 2H). The results showed that bladder urothelial carcinoma (BLCA) patients with TCF3 ^MUT^ experienced poorer DSS ( *P*  = 0.023), while UCEC patients with TCF4 ^MUT^ experienced improved PFS ( *P*  = 0.0035). Moreover, SKCM patients with TCF7 ^MUT^ had worse DSS ( *P*  = 0.015) and OS ( *P*  = 0.025).

### Prognostic prediction of TCF3 for large-scale pan-cancer

**Figure 3:**
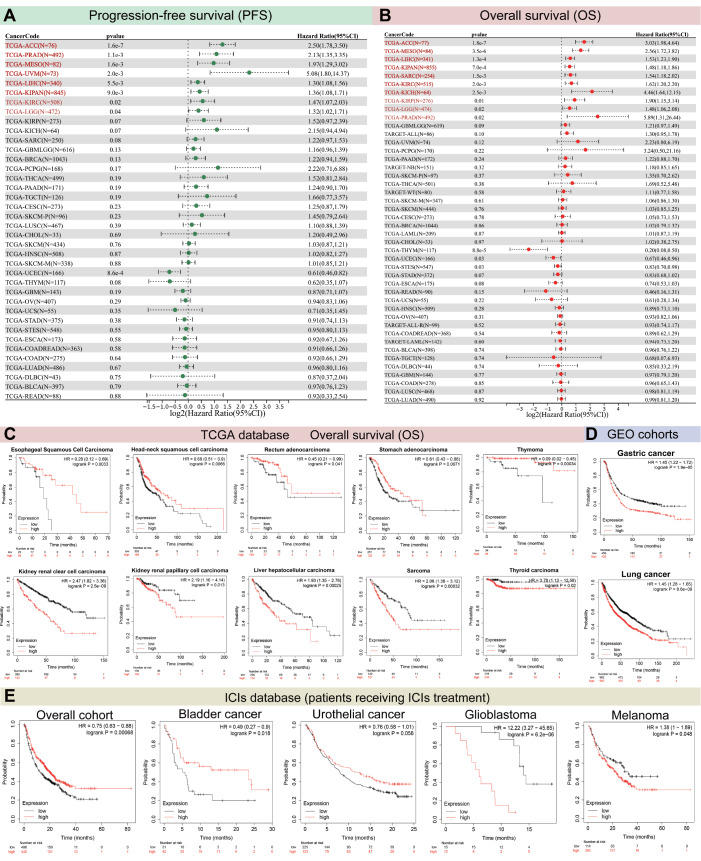
Figure 3 Prognostic ability of TCF3 in a large-scale pancancer dataset (A) Prognostic ability of TCF3 for overall survival in a pancancer cohort. (B) Prognostic ability of TCF3 for progression-free survival in a pancancer cohort. (C) Association of TCF3 expression with overall survival (OS) across distinct tumor types from the TCGA database. (D) Association of TCF3 expression with overall survival (OS) across gastric cancer and lung cancer patients from the GEO cohorts. (E) Association of TCF3 expression with OS in patients treated with ICIs from the ICI database.

After inspecting the mutation landscape, we investigated the impact of TCF3 on pan-cancer PFS and OS ( Figure 3A,B). The forest plot demonstrated a marked prognostic prediction of TCF3 expression in adrenocortical carcinoma (ACC) (OS, *P*  < 0.001; PFS, *P*  < 0.001), prostate adenocarcinoma (PRAD) (OS, *P*  = 0.0011; PFS, *P*  = 0.02), mesothelioma (MESO) (OS, *P*  = 0.0016; PFS, *P *≤ 0.001), uveal melanoma (UVM) (OS, *P*  = 0.002), liver hepatocellular carcinoma (LIHC) (OS, *P*  = 0.0055; PFS, *P*  < 0.001), KIPAN (OS, *P*  = 0.009; PFS, *P*  < 0.001), KIRC (OS, *P*  = 0.02; PFS, *P*  = 0.002), lower grade glioma (LGG) (OS, *P*  = 0.04; PFS, *P*  = 0.02), sarcoma (SARC) (PFS, *P*  = 0.0015), kidney chromophobe (KICH) (PFS, *P*  = 0.0025), and KIRP (PFS, *P*  = 0.01). Next, we performed an in-depth Kaplan-Meier survival analysis to examine the particular impact of TCF3 on patient survival in various cancers. We demonstrated that TCF3 exerted a significant influence on the prognosis of patients with various types of cancers from the TCGA database ( Figure 3C). In esophageal squamous cell carcinoma (HR = 0.28, *P*  = 0.0033), head-neck squamous cell carcinoma (HR = 0.68, *P*  = 0.0066), rectum adenocarcinoma (HR = 0.45, *P*  = 0.041), stomach adenocarcinoma (HR = 0.61, *P*  = 0.0071), and thymoma (HR = 0.09, *P*  = 0.00034), higher levels of TCF3 expression were associated with more favorable patient prognoses. Interestingly, the expression of TCF3 was higher in esophageal squamous cell carcinoma, rectum adenocarcinoma, and stomach adenocarcinoma tissues than in normal tissues ( Figure 2C). Nevertheless, TCF3 also exhibited contrary prognostic implications, such as kidney renal clear cell carcinoma (HR = 2.47, *P*  < 0.001), kidney renal papillary cell carcinoma (HR = 2.19, *P*  = 0.013), liver hepatocellular carcinoma (HR = 1.93, *P*  = 0.00025), sarcoma (HR = 2.08, *P*  = 0.00032), and thyroid carcinoma (HR = 3.78, *P*  = 0.02). In kidney papillary cell carcinoma and liver hepatocellular carcinoma, TCF3 levels were increased, whereas decreased expression of TCF3 was observed in thyroid carcinoma ( Figure 2C). We performed a similar analysis on patients from GDO cohorts ( Figure 3D). Interestingly, the TCF3 expression level was negatively correlated with the prognosis of patients with gastric cancer (HR = 1.45, *P*  < 0.001) and lung cancer (HR = 1.45, *P*  < 0.001). To determine the relationship between TCF3 expression and the prognosis of patients treated with immune checkpoint inhibitors (ICIs), we further analyzed data from the ICI database ( Figure 3E). Elevated expression of TCF3 was linked to better outcomes in the overall cohort (HR = 0.75, *P*  = 0.00068) and in bladder cancer (HR = 0.49, *P*  = 0.018) and urothelial cancer (HR = 0.76, *P*  = 0.056) patients, while glioblastoma (HR = 12.22, *P*  < 0.001) and melanoma (HR = 1.38, *P*  = 0.048) were the opposite. Taken together, these findings indicate that TCF3 has a certain impact on the survival of pan-cancer patients and ICI-treated patients.

### Varied expression and prognostic value of TCF3 in KIRC

**Figure 4:**
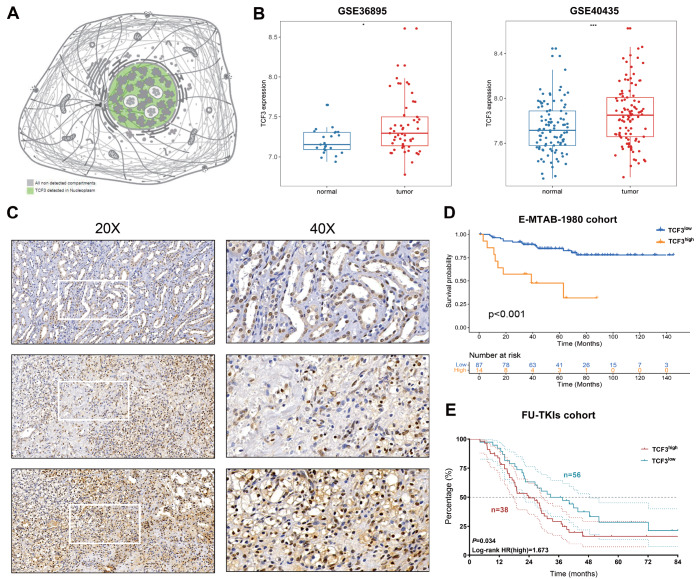
Figure 4 Varied expression and prognostic value of TCF3 in KIRC (A) Pattern diagram of TCF3 expression and localization in cells. (B) Comparison of TCF3 expression between normal and tumor samples from the GSE36895 and GSE40435 datasets. (C) The protein expression of TCF3 in immunohistochemical images of KIRC. (D) Association of TCF3 expression with overall survival (OS) in the E-MTAB-1980 cohort. (E) Association of TCF3 expression with overall survival (OS) in the FU-TKI cohort. *P < 0.05, ***P < 0.001.

As illustrated in Figure 4A, TCF3 was predominantly detected in the nucleoplasm. We assessed the difference in TCF3 expression between normal and tumor samples from the GSE36895 and GSE40435 datasets ( Figure 4B). In both databases, the tumor samples exhibited substantially increased expression of TCF3 ( *P*  < 0.05 in GSE36895, *P*  < 0.001 in GSE40435). To test our hypothesis and predict outcomes derived from bioinformatics, we conducted an IHC analysis of TCF3 to determine its expression pattern in tumor and adjacent normal tissues of KIRC ( Figure 4C). The results demonstrated that TCF3 was mainly expressed in the nucleoplasm and exhibited notably higher staining intensity in tumor tissues. Furthermore, we validated the prognostic significance of TCF3 expression in the E-MTAB-1980 cohort ( *n*  = 101, *P*  < 0.001) and FU-TKI cohort ( *n*  = 94, *P*  = 0.034) ( Figure 4D,E). In both cohorts, high expression of TCF3 was correlated with worse prognosis.

### Association between TCF3 expression and tumor immune infiltration

After clarifying the link between TCF3 expression and tumorigenesis, we subsequently investigated the connection between TCF3 and immune infiltration. Co-expression analyses were carried out across cancers, which clearly revealed that TCF3 was co-expressed with most immune-related genes ( Supplementary Figure S1A,B). Additionally, our CIBERSORT analysis revealed a significant association between TCF3 expression and the level of immune cell infiltration in most cancer types ( Supplementary Figure S1C,D). Specifically, in THYM, the TCF3 expression level was positively related to T-cell regulation (Tregs), T follicular helper cells, naïve CD4 ^+^ T cells, and resting myeloid dendritic cells, while it was negatively correlated with T-cell gamma delta, activated memory CD4 ^+^ T cells, activated NK cells, activated mast cells, M2 macrophages, and M1 macrophages. Ultimately, we assessed the variance in immune checkpoint molecules between groups with high and low TCF3 expression and found that the group with elevated TCF3 expression exhibited significantly increased HAVCR2 ( *P*  < 0.05) and PDCD1LG2 ( *P*  < 0.05) expressions ( Supplementary Figure S1E).

### Correlations of TCF3 expression with heterogeneity, immune scores, angiogenesis, and functional states

**Figure 5:**
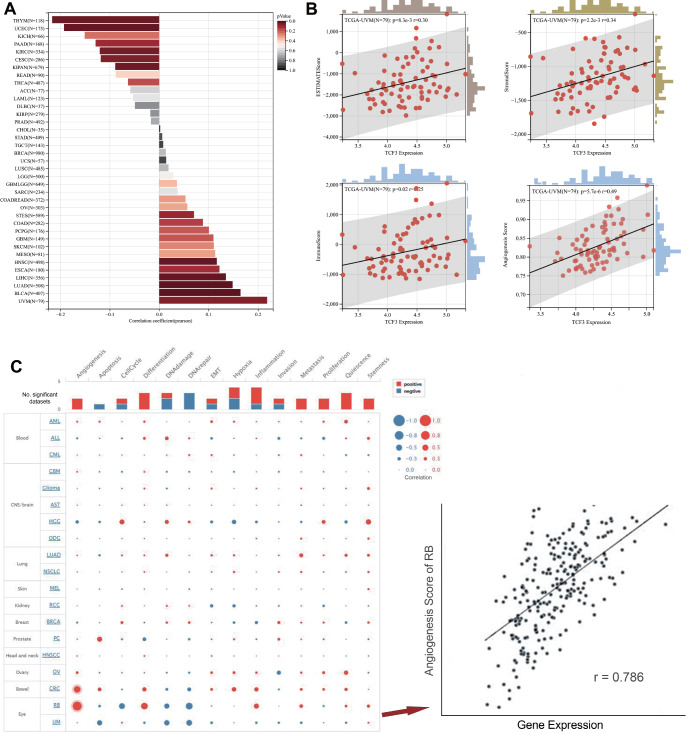
Figure 5 Correlations of TCF3 expression with heterogeneity, immune scores, angiogenesis, and functional states (A) Correlation between heterogeneity and TCF3 expression. (B) Correlations of TCF3 expression with the ESTIMATEScore, StromalScore, ImmuneScore, and angiogenesis according to the RSEM. (C) Correlations between TCF3 expression and 14 cancer functional states across cancers. The right panel represents the correlation between TCF3 expression and angiogenesis score in retinoblastoma (RB) using Pearson’s analysis. The horizontal coordinate represents the amount of TCF3 expression, and the vertical coordinate represents the angiogenesis score in the RB. *P < 0.05, **P < 0.01, ***P < 0.001, ****P < 0.0001.

We conducted an assessment of heterogeneity with the MATH algorithm, as represented in the lollipop-style graph ( Figure 5A). TCF3 demonstrated the most pronounced correlation with heterogeneity in UVM, but in thymoma (THYM), the correlation was inversely related. Then, we independently examined the relationships among TCF3 expression, RSEM expression, three immune scores (ESTIMATEScore, StromalScore, and ImmuneScore) and angiogenesis scores in the UVM using data from the TCGA database ( *P*  = 5.7e–6, r = 0.49; Figure 5B). Interestingly, our analysis revealed a significant positive correlation between TCF3 expression and the following parameters: ESTIMATEScore ( *P*  = 0.0083), StromalScore ( *P*  = 0.0022), ImmuneScore ( *P*  = 0.02), and angiogenesis ( *P*  = 0.001). We further investigated the association between TCF3 and 14 distinct cancer functional states utilizing single-cell sequencing data from CancerSEA ( Figure 5C). The right panel represents the correlation between TCF3 expression and angiogenesis score in retinoblastoma (RB) using Pearson’s analysis, indicating a markedly positive association between TCF3 expression and angiogenesis hallmarks in RB. Our analysis revealed that TCF3 was predominantly positively correlated with differentiation and quiescence across most tumor types, while it was negatively correlated with DNA repair in the majority of tumors.

### Tumor-promoting role of TCF3 *in vitro*

**Figure 6:**
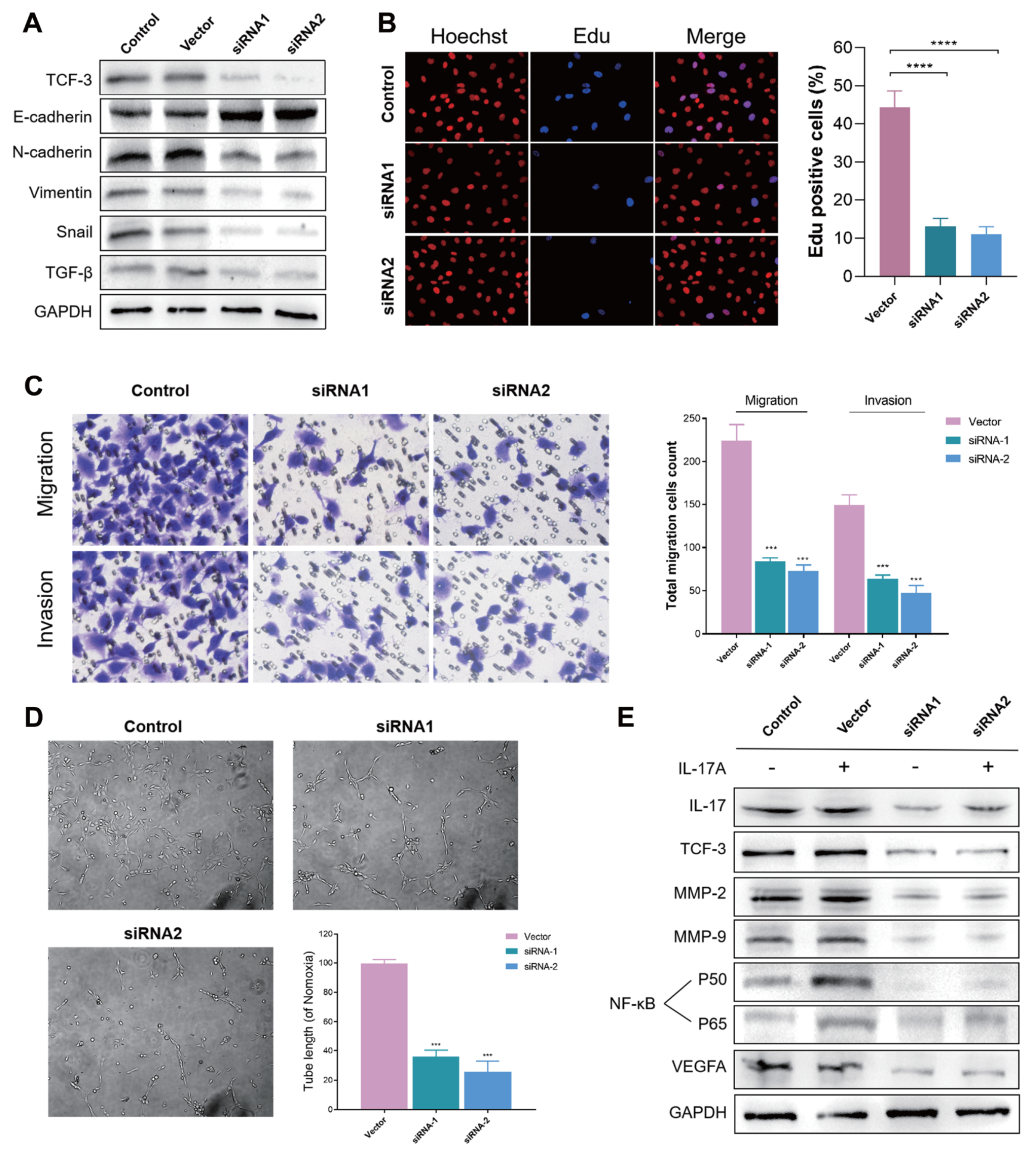
Figure 6 Tumor-promoting role of TCF3 *in vitro* (A) Western blot analysis of TCF3, E-cadherin, N-cadherin, Vimentin, Snail, and TGF-β expressions in MUM2B UVM cells. (B) Immunofluorescence analysis of cells by EdU assay and the proportion of EdU-positive MUM2B cells. (C) Transwell migration and invasion assays using MUM2B cells and the proportion of total migrating cells. (D) Tube formation assay using MUM2B cells and the proportion of tube length. (E) Western blot analysis of IL-17, TCF-3, MMP-2, MMP-9, NF-κB (P50/P65), VEGFA, and GAPDH expressions in MUM2B cells. ***P < 0.001, ****P < 0.0001.

**Figure 7:**
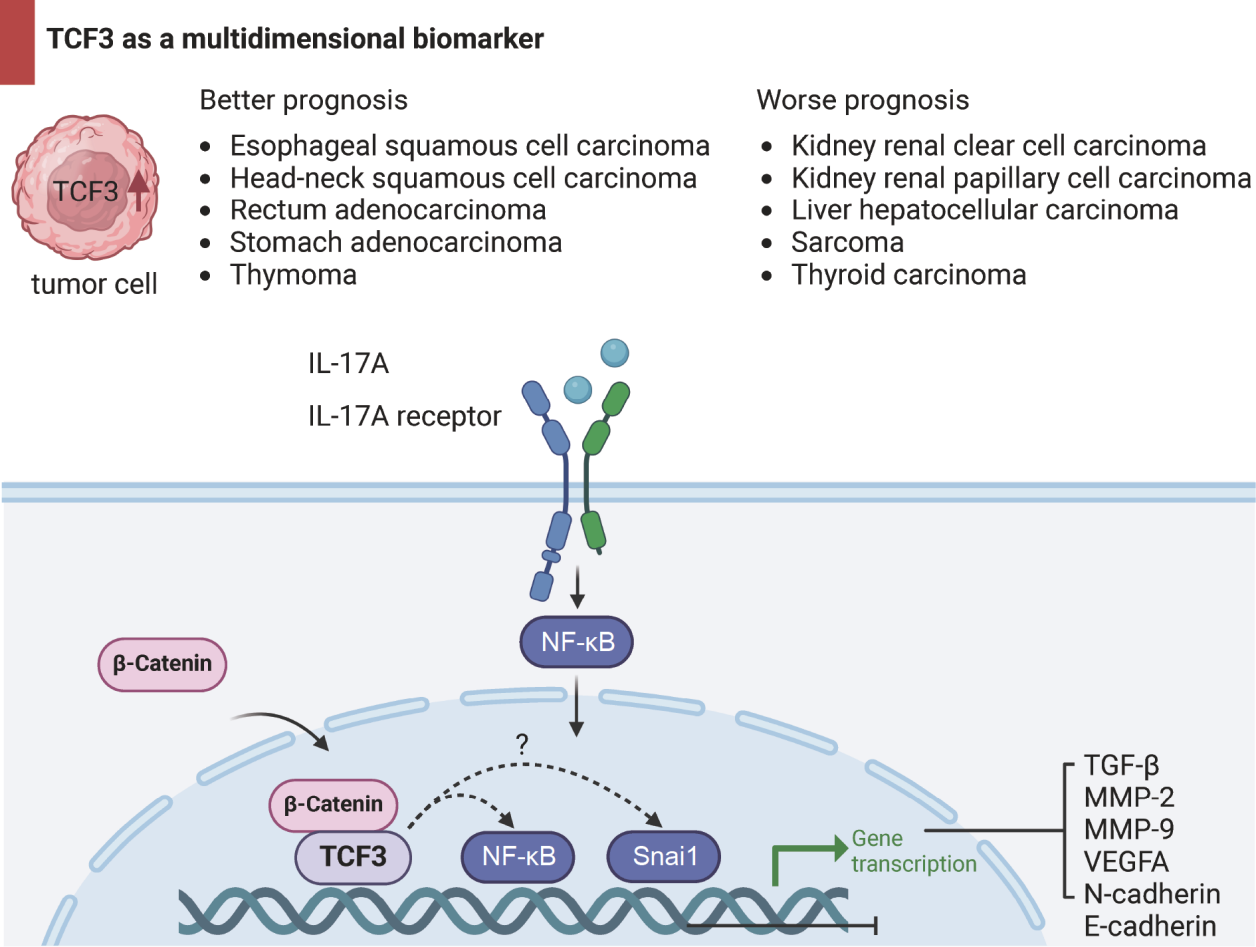
Figure 7 Schematic diagram of the research hypothesis Illustration of the key research highlights derived from the overall findings of this study, ensuring a focus on TCF3 as a multidimensional biomarker.

Furthermore, we investigated the functional significance of TCF3 in MUM2B UVM *in vitro*. We generated *TCF3*-knockdown (siRNA1 and siRNA2) human UVM cells and utilized western blot analysis to confirm the knockdown efficiency ( Figure 6A). It is widely recognized that the process of EMT is a critical factor in the progression of cancer [36]. Therefore, we assessed the levels of proteins associated with EMT ( Figure 6A). The findings revealed an increase in E-cadherin and a decrease in N-cadherin, vimentin, Snail, and TGF-β in *UVN*-knockdown cells. Snail is a group of transcription factors that facilitate the suppression of E-cadherin and influence the EMT process. These changes indicated that TCF3 may play an important role in facilitating the EMT process. Next, to examine the effect of TCF3 on proliferation, we conducted EdU assay and observed a reduction in proliferation in *TCF3*-knockdown cells ( *P*  < 0.0001; Figure 6B). Next, Transwell assays were employed to demonstrate the migratory and invasive capabilities of the cells ( *P*  < 0.001; Figure 6C). *TCF3-*knockdown cells exhibited diminished migration and invasion potential. Moreover, we performed a tube formation assay to evaluate the impact of TCF3 on angiogenesis, which was markedly weakened in *TCF3*-knockdown cells ( *P*  < 0.001; Figure 6D). Collectively, these findings suggested that TCF3 plays a crucial role in promoting various stages of tumor progression, including EMT, migration, invasion, and angiogenesis. To further investigate the underlying biological mechanism, we treated cells with IL-17A and detected downstream proteins by western blot analysis. As shown in Figure 6E, the levels of TCF-3, MMP-2, MMP-9, NF-κB (P50/P65), and VEGFA were lower in the siRNA1 group than those in the control group. Furthermore, the introduction of IL-17A did not reverse this effect due to the absence of *TCF3* in siRNA2-transfected cells. These results suggested that TCF-3 is crucial for modulating IL-17 activity, and the expressions of NF-κB-targeted genes can be increased via the collaboration of the β-catenin:TCF/LEF and NF-κB transcriptional complexes. TCF3 and IL-17 could interact, thereby enhancing downstream activities through the NF-κB signaling pathway. A graphical abstract has been created to illustrate the key research highlights derived from the overall findings of this study, ensuring a focus on TCF3 as a multidimensional biomarker ( Figure 7).

## Discussion

In the development of cancer, the TCF3 protein, a member of the E protein family of nuclear transcription factors, plays a crucial role in the transcriptional regulation of numerous genes. It is actively involved in B lymphocyte formation and contributes significantly to the onset of lymphoma [37]. Additionally, TCF3 participates in the regulation of colon cancer initiation, EMT, metastasis, and pancreatic cancer cell proliferation [ 33, 38]. This highlights the diverse regulatory functions of TCF3 in various aspects of tumorigenesis and cancer progression. Specifically, the characteristics and implications of TCF3 expression patterns have an impact on promoting tumor-related angiogenesis and reshaping the immunosuppressive TME [3].

TCF-3 has been implicated in diverse aspects of tumor development. In colorectal cancer, it participates in tumor initiation and progression by interacting with the tumor microenvironment. Additionally, TCF-3 plays a critical role in B lymphocyte development and lymphomagenesis, highlighting its involvement in hematological malignancies. Its multifaceted roles underscore the complexity of its functions across different tumor types. In this study, we identified distinct TCF3 expression patterns in large-scale cancer cohorts that were strongly associated with differences in the clinicopathological features of the TME. More specifically, we found that significantly upregulated TCF3 expression was correlated with disease progression, long-term prognosis and suppression of immune stimulatory factors in various cancer types. Additionally, our study revealed that the downregulation of TCF3 led to an increase in E-cadherin level and a decrease in N-cadherin, Vimentin, Snail, and TGF-β levels in UVN cells. These changes indicated that TCF3 may play an important role in facilitating the EMT process. TCF3 may promote tumor-associated pathologic angiogenesis, reshape the oxygen-depleted immune-cold tumor microenvironment, and significantly predict poor prognosis and unfavorable responses to VEGF-TKI therapy in patients with cancer, notably in KIRC and UVM. Furthermore, CCL5 has been demonstrated to be a predictive biomarker for evaluating the migration of tumor cells and the EMT pathway, thereby contributing to the aggressive progression of UVM [39]. Consistently, in this study, we showed that an aggressive TCF3 ^high^ phenotype was strongly correlated with the progression of malignancy, migration, and proliferation of tumor cells and rescued the activation of the NK-κB and VEGF signaling pathways after IL-17A induction. These results suggested that TCF-3 plays a crucial role in modulating IL-17 activity. Furthermore, given that the expressions of NF-κB-targeted genes can be enhanced through the collaboration of the β-catenin:TCF/LEF and NF-κB transcriptional complexes, it is possible that TCF3 and IL-17 may interact, thereby amplifying downstream activities through the NF-κB signaling pathway. Therefore, the inability of IL-17 to restore the expressions of NF-κB downstream genes may be associated with this phenomenon. Our findings can contribute to the development of new drugs and targeted treatment strategies for cancer patients associated with pathological angiogenesis [40].

IL-17 is a highly pleiotropic proinflammatory cytokine that is crucial for various processes, including host defense, tissue repair, the pathogenesis of inflammatory diseases, and cancer progression. While activation of the T-cell receptor is key for IL-17 production by CD4 ^+ ^ and CD8 ^+ ^ T cells, innate immune cells primarily generate IL-17 under the influence of inflammatory cytokines. The recognition of TCF-3 as a key player in tumorigenesis opens avenues for therapeutic interventions. Targeting TCF-3 with inhibitors or modulators is an active area of research aiming to disrupt its oncogenic functions. Insights into the molecular mechanisms underlying the effects of TCF-3 could pave the way for the development of novel anticancer strategies, such as strategies targeting the IL-17 pathway. Recent studies have indicated that even under hypoxic conditions, IL-17A signaling through the IL-17RC receptor rapidly induces the ERK/AKT/mTOR pathway, enhancing the transcription and protein expression of HIF1α [ 41, 42]. The IL-17-HIF1α axis guides transcriptional and functional programs related to glycolysis, promoting cell migration.

By directly stimulating cancer cells and indirectly inducing an immunosuppressive tumor microenvironment, IL-17 promotes tumorigenesis. The binding of IL-17 to IL-17R on tumor cells activates downstream molecules, including transcription factors (NF-κB, STAT, and AP-1), kinases (MAPK and HER1), matrix metalloproteinases (MMPs), and anti-apoptotic proteins (Akt, Erk, mTOR, Bcl-2, and Bax). For instance, IL-17 stimulates ovarian cancer stem cell proliferation and self-renewal in a dose-dependent manner through the NF-κB and MAPK pathways [43]. It promotes invasion in hepatocellular carcinoma and epithelial–mesenchymal transition (EMT) in prostate cancer via MMP-2, MMP-7, MMP-9, and NF-κB signaling. IL-17 directly enhances the proliferation of keratinocytes, promotes MMP-dependent cell invasion, supports angiogenesis, inhibits TGF-β-dependent apoptosis, and enhances MEK-, ERK-, JNK-, and STAT3-mediated breast cancer cell proliferation [44]. Consequently, IL-17 is implicated in the tumorigenesis of various cancer types.

IL-17 also shapes the immunosuppressive microenvironment indirectly through chemokines and cytokines, supporting cancer cell proliferation. Specifically, IL-17 induces the expressions of CXCL1, CXCL5, CXCL6, and CXCL8, enhancing myeloid-derived suppressor cell function in breast cancer, suppressing T-cell infiltration in lymphoma models, recruiting tumor-associated macrophages in pancreatic cancer, and supporting angiogenesis and *in vivo* tumor growth in lung cancer [45]. IL-17 upregulates systemic G-CSF expression in breast cancer, attracting immunosuppressive neutrophils and maximizing the potential for breast cancer metastasis. In summary, the direct and indirect oncogenic effects of IL-17 complement each other, stimulating early tumor growth and suppressing the immune system. These findings suggest that IL-17 supports tumor growth, progression, treatment resistance, and metastasis, with a more pronounced role in the early stages of tumor development [46].

We recognize several limitations of this work. First, the retrospective nature of the large-scale cohorts enrolled in this study necessitates future prospective validation. In addition, our research was not able to include in-depth experiments on the mechanism of the pro-tumorigenic role of TCF-3 in cancer and the role of the IL-17/TCF-3/NF-κB/MMP2 axis in regulating aberrant malignancy status, which might reflect potential replicability issues in clustering patients with differential TCF-3 infiltration statuses.

In conclusion, this study is the first to reveal that TCF-3, a member of the TCF/LEF family, plays a significant role in tumor biology and alterations in the TME, with potential clinical implications. Understanding its intricate involvement in tumor development offers opportunities for advancing diagnostic, prognostic, and therapeutic strategies, fostering the prospect of more effective and tailored cancer treatments.

## Supporting information

24156Supplementary_Figure_S1

## Supplementary Data

Supplementary data is available at *Acta Biochimica et Biophysica Sinica* online.
