# Supplementary material for: TCF3 as a multidimensional biomarker: oncogenicity, genomic alterations, and immune landscape in pan-cancer analysis: TCF3 as a pan-cancer multidimensional biomarker
Source: Acta Biochim Biophys Sin (Shanghai). 2024 Aug 27;57(2):195–208. doi: 10.3724/abbs.2024126 (PMC11868920; doi:10.3724/abbs.2024126)
Supplement: 24156Supplementary_Figure_S1 [file 24156Supplementary_Figure_S1.pdf]

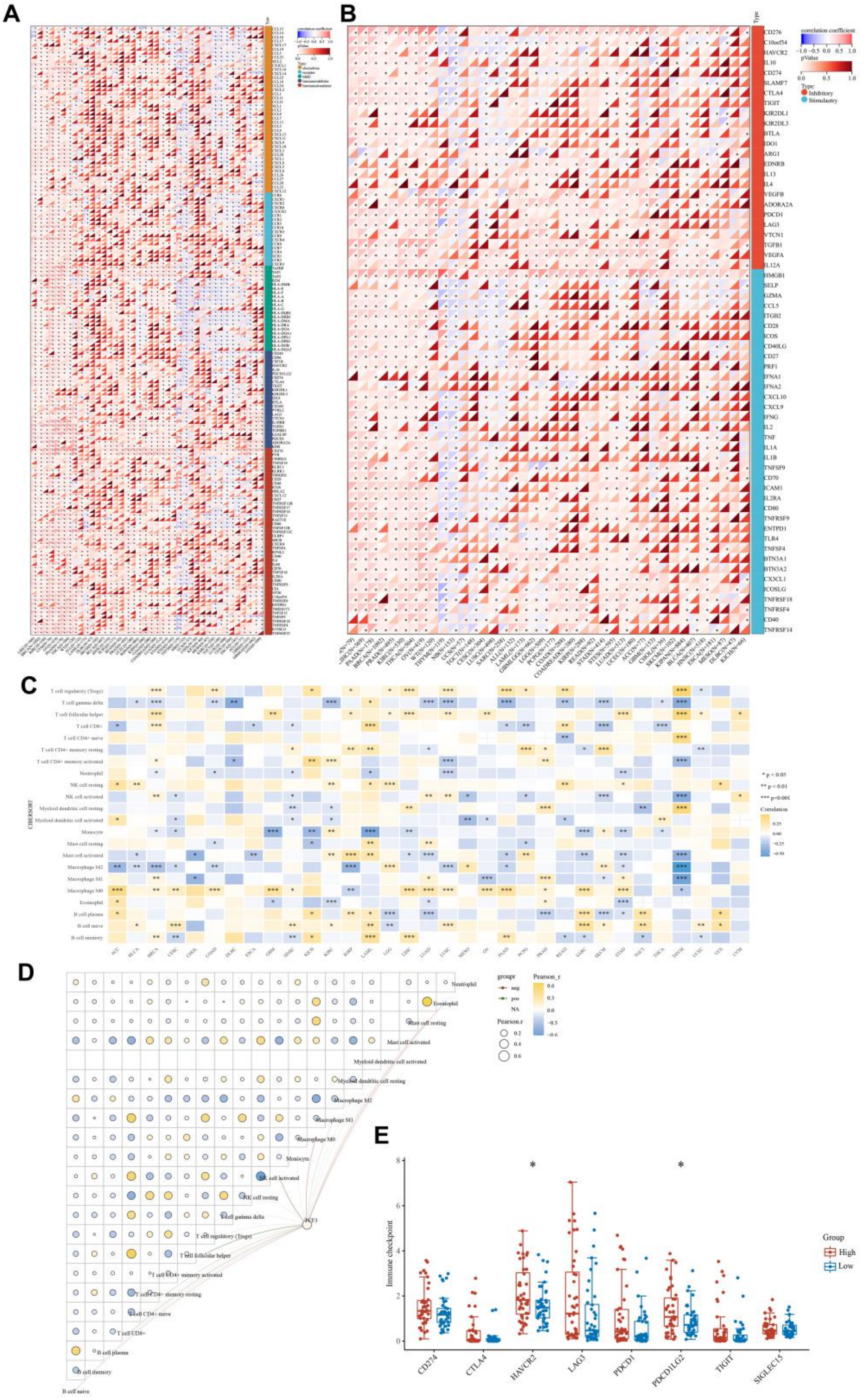

Supplementary Figure S1. Associations between TCF3 expression and tumor immune infiltration (A) Coexpression of TCF3 with chemokines, receptors, MHC, immune inhibitors, and immune stimulators across cancers. (B) Coexpression of TCF3 with immune

inhibitors and immunostimulators across cancers. (C) Relationship between TCF3 expression and immune cell infiltration across cancers. (D) Relationship between TCF3 expression and immune cell infiltration. (E) Expressions of immune checkpoint molecules in the high and low TCF3 expression groups. *P<0.05, **P <0.01, ***P<0.001 and ****P<0.0001.
